# Could setmelanotide be the game-changer for acquired hypothalamic obesity?

**DOI:** 10.3389/fendo.2023.1307889

**Published:** 2024-01-04

**Authors:** Hanneke M. van Santen, Christian Denzer, Hermann Lothar Müller

**Affiliations:** ^1^ Department of Pediatric Endocrinology, Princess Máxima Center for Pediatric Oncology, Utrecht, Netherlands; ^2^ Wilhelmina Children’s Hospital, University Medical center Utrecht (UMCU), Utrecht, Netherlands; ^3^ Division of Pediatric Endocrinology and Diabetes, Department of Pediatrics and Adolescent Medicine, University Medical Center Ulm, Ulm, Germany; ^4^ University Children’s Hospital, Carl von Ossietzky University Oldenburg, Klinikum Oldenburg AöR, Oldenburg, Germany

**Keywords:** hypothalamic obesity, Craniopharyngioma, Setmelanotide, brain tumor, obesity

## Abstract

Children with acquired hypothalamic obesity, e.g. following treatment for pediatric craniopharyngioma are at great risk for metabolic syndrome, cardiovascular health problems and premature mortality. Treatment for acquired hypothalamic obesity has thus far been disappointing. Several interventions were reported to be partially successful, including dextro-amphetamine and GLP-1R agonists, although results in acquired hypothalamic obesity are conflicting. Disruption of signaling through the melanocortin-4 receptor (MC4R) pathway results in hyperphagia and severe early-onset hypothalamic obesity. Recently, the MC4R agonist setmelanotide has shown promising results in children with genetic forms of hypothalamic obesity; POMC, PCSK1 and LEPR. Patient quotes such as “we have our family life back” illustrate the magnitude of the effect. Targeted hormone replacement therapy with a MC4R agonist for acquired hypothalamic obesity could be a game-changer. Preliminary results of setmelanotide treatment in 14, mostly pediatric, patients with acquired hypothalamic obesity are promising. The FDA has recommended that a prospective, randomized, blinded trial be conducted over a 12 months treatment period, comparable to pivotal trials for other obesity drugs. It may be discussed whether setmelanotide should be regarded as an obesity drug or whether it may be envisioned as an agent for hypothalamic substitution therapy. In this commentary we discuss the trial that is currently recruiting patients with acquired hypothalamic obesity.

Obesity is a world-wide pandemic and a challenging problem. When the underlying cause for obesity is hypothalamic syndrome, it is an untreatable and devastating long-term problem (1).

Children with acquired hypothalamic syndrome, e.g. following treatment for a tumor in the hypothalamic region, usually have had an average BMI during childhood. After management of their suprasellar tumor this may change dramatically. Hypothalamic syndrome leads to pituitary deficiencies, disruption of circadian rhythm, disturbed hunger-satiety and thirst feelings, temperature regulation, neurocognitive, sleep and behavioral problems (1). Consequently, patients with hypothalamic syndrome develop (morbid) hypothalamic obesity and chronic fatigue. They are at great risk for metabolic syndrome, cardiovascular health problems and premature mortality.

Treatment for acquired hypothalamic obesity has thus far been disappointing (2). Hypothalamic obesity is a complex, multifaceted disease that may not require a one-size-fits-all approach. In 2019, a personalized treatment algorithm was introduced to improve the management of hypothalamic obesity (2). Several interventions have been reported to be partially successful (1), including dextro-amphetamine (3, 4), somatostatin analoges (5) and GLP-1R agonists, although results in acquired hypothalamic obesity are conflicting.

Disruption of signaling through the melanocortin-4 receptor (MC4R) pathway results in hyperphagia and severe early-onset hypothalamic obesity. Recently, the MC4R agonist setmelanotide has shown promising results in children with mutations in the leptin-melanocortin pathway genes (6).

It may be questioned whether an MC4R agonist would also work for children with acquired hypothalamic obesity. If the hypothalamus is damaged, or completely resected, how can it work? In many cases with acquired hypothalamic obesity, not the entire hypothalamus is lost and thus patients may have a partial hypothalamic dysfunction. Setmelanotide could therefore activate the remaining hypothalamic neurons. Secondly, MC4 receptors have been also shown to be expressed in the cerebral cortex and spinal cord (7).

In a child with proven pituitary dysfunction, there is no discussion of the need for hormone replacement therapy. Treatment with hydrocortisone, thyroid hormone, sex steroids, growth hormone, and vasopressin is routinely and rapidly initiated. However, hypothalamic obesity, that often aggravates with astonishing speed in the first year after surgery, cannot be adequately controlled. Dietary and lifestyle counseling is provided, and obesity management education is started.

One could state that if the MC4R does not properly integrate satiety signals, for whatever reason, this resembles a state of leptin deficiency. Restoration of leptin in patients with leptin deficiency normalizes BMI. In this light, we may consider treatment with setmelanotide in patients with hypothalamic obesity as “restoring” the hypothalamic satiety signaling, comparable to restoring a neuroendocrine deficiency. This would imply that setmelanotide treatment would in fact be hormonal substitution therapy and not obesity treatment per se.

Targeted hormone replacement therapy with an MC4R agonist for acquired hypothalamic obesity could be a game-changer. Preliminary results of setmelanotide treatment in 14, mostly pediatric, patients with acquired hypothalamic obesity are promising (https://clinicaltrials.gov/ct2/show/NCT04725240). After 16 weeks of treatment, impressive changes in BMI and, perhaps most importantly, significant effects on hyperphagia and quality of life for the patient and family have been observed (8). Patient quotes such as “we have our family life back” illustrate the magnitude of the effect (personal communication). The FDA has now recommended a prospective, randomized, blinded trial over a 12 months treatment period, comparable to other obesity interventions (https://clinicaltrials.gov/ct2/show/NCT05774756). While this may be the best way to study its efficacy, we question whether this trial is feasible with children having to inject themselves with placebo for one year. If such a trial “fails”, due to attrition in the control population, we would strongly argue for continuing with the evaluation of the intervention group. To study the effect of setmelanotide as if it were an anti-obesity drug may not be necessary; it may be envisioned as hypothalamic substitution therapy.

If the planned trial is successful, we must develop a new personalized treatment algorithm for hypothalamic obesity. We envision future cohorts of patients with acquired hypothalamic obesity, with standardized diagnostics, risk stratification and structured prospective evaluation of multimodal treatment approaches including pituitary hormone replacement therapy and combined pharmacological interventions for hypothalamic obesity (e.g. MC4R agonists with a GLP-1 agonist and/or with stimulant treatment). This could lead to the much needed improvements in health outcomes and quality of life in a suffering and underserved patient population.

We will (impatiently and hopefully) await the results of the prospective study with setmelanotide in patients with acquired hypothalamic obesity to learn about its efficacy, safety and tolerability.

## Data availability statement

The original contributions presented in the study are included in the article/supplementary material. Further inquiries can be directed to the corresponding author.

## Author contributions

HvS: Conceptualization, Writing – original draft, Writing – review & editing. CD: Conceptualization, Writing – original draft, Writing – review & editing. HM: Conceptualization, Writing – original draft, Writing – review & editing.

## References

[1] Müller HL Tauber M Lawson EA Özyurt J Bison B Martinez-Barbera JP. Nat Rev Dis Prim (2022) 8:24. doi: 10.1038/s41572-022-00351-z 35449162

[2] van Iersel L Brokke KE Adan RAH Bulthuis LCM van den Akker ELT van Santen HM. Pathophysiology and individualized treatment of hypothalamic obesity following craniopharyngioma and other suprasellar tumors: A systematic review. Endocr. Rev (2018) 40:193–235. doi: 10.1210/er.2018-00017 30247642

[3] Denzer C Denzer F Lennerz BS Vollbach H Lustig RH Wabitsch M. Treatment of hypothalamic obesity with dextroamphetamine: A case series. Obes Facts (2019) 12:91–102. doi: 10.1159/000495851 30844799 PMC6465734

[4] van Schaik J Welling MS de Groot CJ van Eck JP Juriaans A Burghard M. Dextroamphetamine treatment in children with hypothalamic obesity. Front Endocrinol (2022) 13:845937. doi: 10.3389/fendo.2022.845937 PMC8959487 35355559

[5] Lustig RH Rose SR Burghen GA Velasquez-Mieyer P Broome DC Smith K. Hypothalamic obesity caused by cranial insult in children: altered glucose and insulin dynamics and reversal by a somatostatin agonist. J Pediatr (1999) 135:162–8. doi: 10.1016/S0022-3476(99)70017-X 10431109

[6] Clément K van den Akker E Argente J Bahm A Chung WK Connors H. Efficacy and safety of setmelanotide, an MC4R agonist, in individuals with severe obesity due to LEPR or POMC deficiency: single-arm, open-label, multicentre, phase 3 trials. Lancet Diabetes Endocrinol (2020) 8:960–70. doi: 10.1016/S2213-8587(20)30364-8 33137293

[7] Siljee-Wong JE. Melanocortin MC4 receptor expression sites and local function. Eur J Pharmacol (2011) 660(1):234–40. doi: 10.1016/j.ejphar.2010.10.104 21199645

[8] Roth CL Shoemaker AH Gottschalk M Miller JL Yuan G Chen E. Special Issue: Abstracts from the 40th Annual Meeting of the Obesity Society at Obesity week, November 1–4, 2022. Obesity (2022) 30(S1):125. doi: 10.1530/endoabs.90.P134

